# Regional wall motion abnormality at the lateral wall disturbs correlations between tissue Doppler E/e′ ratios and left ventricular diastolic performance parameters measured by invasive methods

**DOI:** 10.1007/s12574-013-0193-x

**Published:** 2013-09-26

**Authors:** Toshiharu Fujii, Koichiro Yoshioka, Masataka Nakano, Gaku Nakazawa, Mari Amino, Naoki Masuda, Norihiko Shinozaki, Shigetaka Kanda, Nobuhiko Ogata, Yoshiaki Deguchi, Fuminobu Yoshimachi, Yuji Ikari

**Affiliations:** Department of Cardiology, Tokai University School of Medicine, 143 Shimokasuya, Isehara, 259-1193 Japan

**Keywords:** Heart failure with normal left ventricular ejection fraction, Diastolic phase of mitral annulus velocity, E/e′, Isovolumetric relaxation time constant, Left ventricular end-diastolic pressure, Tissue Doppler imaging

## Abstract

**Background:**

The impact of regional wall motion abnormality (RWMA) on the accuracy of heart failure with preserved ejection fraction (HFpEF) diagnosis using the E/e′ ratio, which is a non-invasive parameter of left ventricular diastolic performance, is unknown. The purpose of this study was to elucidate the impact of RWMA of the lateral wall (RWMAlat) on the correlation between E/e′ and invasive parameters of left ventricular diastolic performance.

**Methods:**

Three hundred and eight consecutive patients undergoing tissue Doppler imaging and catheterization pressure examination were retrospectively analyzed. E/e′ was calculated as the ratio of early diastolic transmitral flow velocity to mitral annular velocity at the lateral wall. Invasive parameters including left ventricular end-diastolic pressure (LVEDP) and isovolumetric relaxation time constant (*τ*) were assessed based on the left ventricular pressure study. Correlation coefficients between E/e′ and these invasive parameters were analyzed and compared between cases with RWMAlat and without RWMA.

**Results:**

LVEDP and *τ* correlated well with E/e′ for all 308 patients (*r* = 0.51 and *r* = 0.65, respectively). Sixty-two patients had RWMA; the remaining 246 did not have RWMAlat. We confirmed that the presence of RWMAlat weakens both the correlations between E/e′ and LVEDP (*r* = 0.574 vs. *r* = 0.381), and E/e′ and *τ* (*r* = 0.729 vs. *r* = 0.461).

**Conclusions:**

Although E/e′ correlates well with parameters of left ventricular diastolic performance assessed by invasive methods, the presence of RWMAlat worsens this correlation. In cases with RWMAlat, careful assessment is required for HFpEF diagnosis because the diagnostic value of the E/e′ ratio could be decreased compared to patients without RWMAlat.

## Introduction

The precise understanding of clinical situations of diastolic heart failure has proven difficult because the diagnostic criteria have not been standardized [1–6]. Recent reports showed that heart failure with preserved ejection fraction (HFpEF) accounts for nearly half of all heart failure cases [7–9]. These reports impressed upon us the need for a non-invasive parameter that reflects left ventricular diastolic performance for standardized diagnostic criteria of HFpEF.

The E/e′ ratio, which is obtained by measurement of the early diastolic transmitral flow velocity (E) and the mitral annular velocity at the early diastolic phase on tissue Doppler (e′), was reported to be a good index for estimating the left ventricular filling pressure [10–15]. E/e′ correlates well with parameters assessed by invasive techniques, such as left ventricular diastolic pressure (LVEDP), which is an important indicator of diastolic dysfunction, or the isovolumetric relaxation time constant (*τ*), which is a parameter reflecting left ventricular relaxation [16–20]. Therefore, the E/e′ ratio is one of the most important diagnostic parameters used by many working groups [6, 21–23].

However, the specificity of E/e′ measurements, based on assessing a spot on the lateral or septal wall, is influenced by factors that can reduce the strength of the correlation between the results of invasive versus non-invasive techniques [17, 24]. In particular, we hypothesized that regional wall motion abnormality (RWMA) on echocardiography contributes to this, which may be problematic when diagnosing HFpEF using tissue Doppler imaging (TDI) in clinical practice.

To study the effect of RWMA at the lateral wall (RWMAlat) on correlations between E/e′ values measured at the lateral wall (E/e′lat) and parameters assessed by invasive techniques (LVEDP and *τ*), we performed correlation analysis and compared correlation coefficients in patients with or without RWMAlat.

## Materials and methods

### Study population

We retrospectively studied the medical records of 308 patients who underwent coronary angiography and left ventricular pressure examination from April 2009 to April 2010 at Tokai University School of Medicine because of suspicion of coronary artery disease or for the evaluation of global cardiac function. These patients had undergone echocardiography and blood testing 2 months prior to the catheterization study. All patients gave written informed consent; this study was designed in accordance with the ethical standards of the General Clinical Research Center of Tokai University School of Medicine.

Exclusion criteria were as follows: low left ventricular ejection fraction (LVEF) below 30 %, abnormal sinus rhythm (atrial fibrillation, atrial flutter, and frequent premature beat), waveform abnormalities (bundle branch block, pacing rhythm), severe mitral annular calcification, and decompensated heart failure in the acute phase.

### Study protocols

To study the impact of RWMAlat on the correlation between E/e′lat values and parameters assessed by invasive techniques, we performed the two following analyses:

1. A correlation between E/e′lat and invasive parameters
  To confirm the correlation between E/e′lat and invasive parameters for all 308 patients, we applied Pearson’s correlation coefficient testing. Invasive parameters also included systolic aortic pressure (AoSP), diastolic aortic pressure (AoDP), maximum rate of left ventricular pressure rise (positive d*p*/d*t*), and peak rate of fall in left ventricular pressure (negative d*p*/d*t*) in addition to LVEDP and *τ*.
2. Impact of RWMAlat on the correlation between E/e′ and invasive parameters
  To confirm the impact of RWMAlat on the correlation between E/e′lat and invasive parameters (LVEDP and *τ*), the correlation coefficients between E/e′ and these parameters were compared in patients with RWMAlat and those without RWMA on echocardiographic assessment.

### Echocardiography

All echocardiographic studies were performed in the supine position using Xario XG with a 1.8–4.2-MHz PST-25AT transducer (Toshiba Medical Systems Corporation, Tochigi, Japan) by an experienced investigator. Comprehensive echocardiographic examination consisted of conventional 2-dimensional, Doppler, and color flow imaging, and TDI.

#### Conventional measurements (2-dimensional, M-mode, pulsed Doppler, color flow imaging)

The recorded echocardiographic data were evaluated and assessed based on the recommendations of the American Society of Echocardiography [25].

For 2-dimensional measurements, the LVEF was calculated in apical 4-chamber and apical 2-chamber views at end-diastole and at end-systole by modified Simpson’s rule [25, 26]. Papillary muscles were excluded from the cavity in the tracking. End-diastole was defined at the onset of the QRS wave on electrocardiographic monitoring, and end-systole was defined as the time of the frame preceding mitral valve opening.

Regional left ventricular function was assessed by a 17-segment model recommended by the American Society of Echocardiography in a parasternal long-axis, 2-chamber, and 4-chamber orientation, and 3 different levels in short-axis orientation [25]. RWMA was classified into 5 categories according to regional left ventricular function as follows: “normal”, “hypokinesis”, “akinesis”, “dyskinesis”, and “aneurysmal”. RWMAs were divided into 3 categories by their location, i.e., (1) anterior, antero-septal, or apical cap, (2) inferior, and (3) lateral, infero-lateral, or antero-lateral. These 3 categories were designated the “antero-septal area”, “inferior area”, and “lateral area”, respectively. Therefore, the RWMAlat was defined as hypokinesis, akinesis, dyskinesis, or aneurysmal in lateral, infero-lateral, or antero-lateral areas.

In M-mode recording, septal wall thickness, posterior wall thickness, and left ventricular internal dimensions were measured over several cardiac cycles in the parasternal short-axis acoustic window to optimize medial–lateral beam orientation. These parameters were measured at the level of the mitral valve leaflet tips at the left ventricular minor axis. The measurements were confirmed by the 2-dimensional method. The thickness of the ventricular wall and chamber size were measured as the distance between the leading edge echoes.

In pulsed-wave Doppler echocardiography, the left ventricular inflow velocity pattern, which consists of early diastolic velocity (E) and late diastolic velocity (A), was recorded while the sample volume was placed at the tip of anterior and posterior mitral leaflets.

In color flow imaging, the severity of valve regurgitation was evaluated based on the recommendations of the American Society of Echocardiography [27, 28]. Regurgitation severity was classified into 3 grades: mild, moderate, and severe, based on the American College of Cardiology/American Heart Association Task Force on Practice Guidelines [29].

#### Tissue Doppler imaging

TDI of the mitral annulus velocity was obtained from the apical 4-chamber view, while a 5- to 10-mm sample volume was placed at the lateral mitral annulus. The diastolic phase of the mitral annulus velocity consists of the early diastolic phase (e′) and the late diastolic phase (a′) as the left ventricular inflow velocity pattern in pulsed-wave Doppler echocardiography. Here, e′ was expressed as E/e′ by calculating the ratio of E to e′.

### Coronary angiography and left ventricular pressure investigations

For coronary angiography and left ventricular pressure assessment, a suitable catheter was inserted via either the femoral or radial artery. Coronary angiography was performed by a 4Fr coronary catheter, which was selected to fit each patient’s coronary arteries, and lesions were evaluated based on the American Heart Association classification [30]. Left ventricular pressure was measured using a 4Fr fluid-filled pigtail catheter. All pressure data were measured and analyzed by an RMC-3000 instrument (Nihon Kohden, Tokyo, Japan). LVEDP, positive d*p*/d*t*, negative d*p*/d*t*, and *τ* were measured. Positive d*p*/d*t* and negative d*p*/d*t* were defined as the maximum and minimum differential values calculated from the following formula:

$$ \left( {{\text{Positive}}/{\text{negative}}} \right)   {\text{dp}}/{\text{dt}} = \frac{{\left[ {{\text{P}}\left( {\text{n}} \right) - {\text{P}}\left( {{\text{n}} - 1} \right)} \right]}}{\text{Sample rate}} $$

*τ* was derived from the slope of the graph (−1/*τ*) plotted as a natural logarithm of the pressure scale on the *y*-axis and time scale on the *x*-axis, which was described by the following mono-exponential equation [31–36]:

$$ P_{{({\text{t}})}} = P_{0} e^{{\frac{{ - {\text{t}}}}{{{\text{P}}{\varvec{\tau}}}}}} + P_{\infty } $$

where *t* is the time from negative d*p*/d*t*, *P*_0_ the mono-exponential amplitude coefficient, *P*_*τ*_ the mono-exponential time constant, and *P*_∞_ is the mono-exponential asymptotic equation.

AoDP and AoSP were also measured after pulling out the catheter from the left ventricle to the ascending aorta. LVEDP, AoDP, and AoDP were recorded as average values of measurements over 3 s. All measurements were recorded while the patients were holding their breath in end-expiratory cycle.

### Statistical analysis

All data are presented as the mean ± standard deviation (SD). Pearson’s correlation coefficient analysis and simple regression were used to assess the associations between invasive or non-invasive measurements. *P* < 0.05 was considered statistically significant. All statistical calculations were performed using JMP version 9 (SAS Institute, Inc., Cary, North Carolina, USA).

## Results

To study the impact of RWMAlat on the correlation between E/e′lat values and parameters assessed by invasive methods (LVEDP and *τ*), we compared this correlation coefficient in patients with or without RWMAlat. Ninety-two patients were excluded according to exclusion criteria (30 patients of low LVEF below 30 %, 9 patients of abnormal sinus rhythm, 34 patients of waveform abnormalities, 12 patients severe mitral annular calcification, and 7 patients of decompensated heart failure), and three hundred and eight patients were analyzed in the present study. The baseline characteristics for all 308 enrolled patients are shown in Table 1. The mean age was 64.1 ± 10.9 years and 82.6 % were male. Table 2 shows the echocardiographic findings. The mean E/e′lat was 12.4 ± 4.4. Fifty-nine percent (*n* = 183) showed no RWMA, and are regarded as controls for the 41 % (*n* = 125) who had RWMA at either area. Among these patients with RWMA, 50 % (*n* = 62) had RWMAlat. Table 3 shows the results of the catheterization study. The mean LVEDP and *τ* were 1.4 ± 24.9 mmHg and 40.1 ± 19.1 ms, respectively.

**Table 1:** Baseline patient characteristics (*n* = 308)

Age (years)	64.1 ± 10.9
Male (%)	82.8 (*n* = 255)
Height (cm)	162.5 ± 9.04
Weight (kg)	62.9 ± 11.5
Body surface area (m^2^)	1.67 ± 0.18
Serum creatinine (mg/dl)	1.45 ± 1.89
Estimated GFR (ml/min)	61.1 ± 23.1
BNP (pg/ml)	96.9 ± 182.5
Hemodialysis (%)	7.5 (*n* = 23)
Diabetes mellitus (%)	36.4 (*n* = 112)
Untreated	8.1 (*n* = 25)
Oral medication	16.9 (*n* = 52)
Insulin	11.4 (*n* = 35)
Current smoker (%)	48.1 (*n* = 148)
Hypertension (%)	74.7 (*n* = 230)
Dyslipidemia (%)	65.6 (*n* = 202)
Total cholesterol (mg/dl)	178.3 ± 41.3
HDL cholesterol (mg/dl)	56.4 ± 18.1
Triglyceride (mg/dl)	144.4 ± 135.9
Prior CVD (%)	10.4 (*n* = 32)
Prior heart failure (%)	10.4 (*n* = 32)
Prior ischemic heart disease (%)	60.1 (*n* = 185)
Prior AP	17.2 (*n* = 53)
Prior UA	7.5 (*n* = 23)
Prior MI	35.4 (*n* = 109)
Dilated cardiomyopathy (%)	1.9 (*n* = 6)
Hypertrophic cardiomyopathy (%)	2.6 (*n* = 8)
Vasospastic angina (%)	4.2 (*n* = 13)
Idiopathic ventricular fibrillation (%)	0.6 (*n* = 2)

*BNP* brain natriuretic peptide, *CVD* cerebrovascular disease, *AP* angina pectoris, *UA* unstable angina, *MI* myocardial infarction

**Table 2:** Echocardiographic findings

LVEF (%)	62.6 ± 12.6
IVS-WT (mm)	11.1 ± 2.1
PWT (mm)	10.3 ± 1.9
LVDd (mm)	50.2 ± 7.0
LVDs (mm)	31.7 ± 8.0
E (cm/s)	56.4 ± 18.4
A (cm/s)	70.4 ± 18.8
E/A	0.8 ± 0.3
e′ (cm/s)	5.1 ± 2.2
E/e′	12.4 ± 4.4
Mitral regurgitation (%)	
Mild	41.6 (*n* = 128)
Moderate	9.1 (*n* = 28)
Severe	0.3 (*n* = 1)
Aortic regurgitation (%)	
Mild	20.8 (*n* = 64)
Moderate	5.8 (*n* = 18)
Severe	1.3 (*n* = 4)
Tricuspid regurgitation (%)	
Mild	34.4 (*n* = 106)
Moderate	3.6 (*n* = 11)
Severe	0.6 (*n* = 2)
Pulmonary regurgitation (%)	
Mild	27.6 (*n* = 85)
Moderate	1.3 (*n* = 4)
Severe	0.3 (*n* = 1)
Aortic stenosis (%)	
Mild	3.2 (*n* = 10)
Moderate	1.6 (*n* = 5)
Severe	0.3 (*n* = 1)
Mitral stenosis (%)	
Mild	0.3 (*n* = 1)
Moderate	0
Severe	0
RWMA at antero-septal area (%)	
Hypokinesis	18.5 (*n* = 57)
Akinesis	2.2 (*n* = 7)
Dyskinesis	2.3 (*n* = 10)
Aneurysmal	0
RWMA at inferior area (%)	
Hypokinesis	21.4 (*n* = 66)
Akinesis	0.6 (*n* = 2)
Dyskinesis	0
Aneurysmal	0
RWMA at lateral area (%)	
Hypokinesis	20.1 (*n* = 62)
Akinesis	0
Dyskinesis	0
Aneurysmal	0

*LVEF* left ventricular ejection fraction, *IVS-WT* interventricular septal wall thickness, *PWT* posterior wall thickness, *LVDd* left ventricular end-diastolic diameter, *LVDs* left ventricular systolic diameter, *E* early diastolic filling velocity, *A* atrial filling velocity, *e′* mitral annulus velocity consisting of the early diastolic phase, *RWMA* regional wall motion abnormality

**Table 3:** Results of right cardiac catheterization and coronary angiography

Significant stenosis (%)	
Right coronary artery	10.7 (*n* = 33)
Left anterior descending artery	17.2 (*n* = 53)
Left circumflex artery	11.4 (*n* = 35)
Left main trunk	1.3 (*n* = 4)
Total occlusion (%)	
Right coronary artery	2.9 (*n* = 9)
Left anterior descending artery	4.2 (*n* = 13)
Left circumflex artery	3.9 (*n* = 12)
Left main trunk	0
Results of the left cardiac pressure study (%)	
LVSP (mmHg)	133.5 ± 25.4
LVEDP (mmHg)	1.4 ± 24.9
AoSP (mmHg)	137.2 ± 24.9
AoDP (mmHg)	65.0 ± 12.1
Positive d*p*/d*t* (mmHg/s)	1571.5 ± 408.9
Negative d*p*/d*t* (mmHg/s)	1506.2 ± 408.2
*τ* (ms)	40.1 ± 19.1

*LVSP* left ventricular systolic pressure, *LVEDP* left ventricular end-diastolic pressure, *AoSP* systolic aortic pressure, *AoDP* diastolic aortic pressure, *Positive dp/dt* maximum rate of left ventricular pressure rise, *Negative dp/dt* peak rate of fall in left ventricular pressure, *τ* isovolumetric relaxation time constant

### Correlations between E/e′lat and invasive parameters

**Fig. 1:**
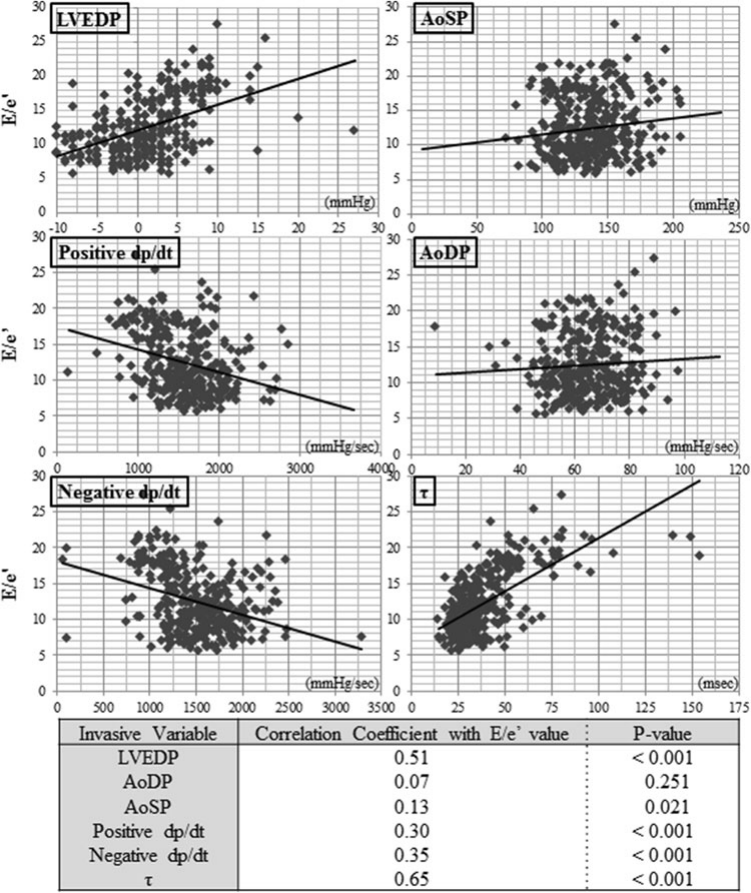
Correlations between the E/e′ ratio and 6 invasive parameters. The correlation coefficients between the tissue Doppler E/e′ ratio and 6 invasive parameters obtained from left ventricular pressure assessments in all 308 patients are shown. Left ventricular end-diastolic pressure (LVEDP) and *τ* correlate well with the value of E/e′

To confirm the correlations between E/e′lat and invasive parameters (LVEDP, AoSP, AoDP, positive d*p*/d*t*, negative d*p*/d*t*, and *τ*), we analyzed the data for all 308 patients (Fig. 1). LVEDP and *τ* correlated well with E/e′lat (*r* = 0.51; *P* < 0.001, and *r* = 0.65; *P* < 0.001, respectively), but the other parameters tested (AoDP, AoSP, positive d*p*/d*t*, and negative d*p*/d*t*) correlated poorly. Moreover, e′ correlated well with *τ* (*r* = 0.46; *P* < 0.001).

### Impact of RWMAlat on the correlation between E/e′lat and invasive parameters

**Fig. 2:**
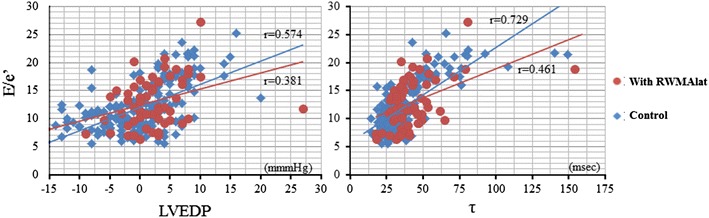
The correlation coefficients between the E/e′ ratio and invasive parameters are influenced by regional wall motion abnormality at the lateral wall. The effect of regional wall motion abnormality at the lateral wall (RWMAlat) on the strength of the correlation between E/e′ and invasive parameters, as seen by the comparison with a control group without RWMAlat, is shown. The E/e′ ratio and LVEDP correlation coefficient of the group with RWMAlat was 0.381 compared to 0.574 for controls with RWMA (*left panel*). Similarly, the E/e′ ration and *τ* correlation coefficient decreased from 0.729 to 0.461 (*right panel*)

To investigate whether RWMAlat affected the good correlation between E/e′lat and LVEDP and *τ*, we compared 62 patients with RWMAlat and 183 controls patients without RWMA (Fig. 2). The results show that the correlation coefficients were low in the group with RWMAlat for both LVEDP and *τ* (left panel; *r* = 0.574 vs. *r* = 0.381, right panel; *r* = 0.729 vs. *r* = 0.461, respectively). Thus, it was confirmed that RWMAlat weakens the correlation between E/e′lat and LVEDP or *τ* of left ventricular diastolic performance parameters. Table 4 shows a comparison of the baseline parameters between patients with and without RWMAlat. The RWMAlat group tended to have low systolic performance, such as low LVEF, high brain natriuretic peptide levels, and enlarged left ventricular chambers. These differences might be a reflection of prior ischemic disease with RWMA.

**Table 4:** Comparison of baseline parameters between patients with and without RWMAlat

	RWMAlat (*n* = 62)	Without RWMAlat (*n* = 246)	*P* value
Age (years)	62.2 ± 12.6	64.6 ± 10.5	0.1245
Male (%)	83.9 (*n* = 52)	82.5 (*n* = 203)	0.8012
Height (cm)	163.5 ± 7.8	162.3 ± 9.3	0.3345
Weight (kg)	62.5 ± 12.3	63.0 ± 11.3	0.7294
Body surface area (m^2^)	1.67 ± 0.18	1.67 ± 0.18	0.9242
Serum creatinine (mg/dl)	1.38 ± 1.75	1.47 ± 1.92	0.7332
Estimated GFR (ml/min)	60.7 ± 22.6	61.3 ± 23.2	0.8544
BNP (pg/ml)	138.1 ± 170.3	85.3 ± 184.6	0.0476
Diabetes mellitus (%)	37.1 (*n* = 23)	36.2 (*n* = 89)	0.8932
Hypertension (%)	74.2 (*n* = 46)	74.8 (*n* = 184)	0.9222
Dyslipidemia (%)	61.3 (*n* = 38)	66.7 (*n* = 164)	0.4258
Total cholesterol (mg/dl)	174.2 ± 34.7	179.3 ± 42.8	0.3869
Triglyceride (mg/dl)	120.0 ± 68.5	150.6 ± 147.7	0.1144
LVEF (%)	50.3 ± 11.0	65.8 ± 10.6	<0.001
IVS-WT (mm)	11.2 ± 2.1	11.0 ± 2.1	0.6079
PWT-WT (mm)	9.8 ± 2.4	10.4 ± 1.7	0.0335
LVDd (mm)	54.6 ± 7.5	49.1 ± 6.4	<0.001
LVDs (mm)	39.4 ± 9.4	29.7 ± 6.3	<0.001
E (m/s)	53.2 ± 21.3	57.3 ± 17.6	0.1191
A (m/s)	65.4 ± 21.7	71.6 ± 17.8	0.0215
E/A	0.85 ± 0.3	0.83 ± 0.3	0.5909
e′ (cm/s)	4.6 ± 2.3	5.1 ± 2.1	0.0801
E/e′	12.9 ± 4.4	12.3 ± 4.4	0.3754
LVEDP (mmHg)	1.85 ± 5.8	0.86 ± 5.9	0.3257
*τ* (ms)	42.3 ± 19.3	39.5 ± 19.1	0.3122

*GFR* glomerular filtration rate, *BNP* brain natriuretic peptide, *LVEF* left ventricular ejection fraction, *IVS-WT* interventricular septal wall thickness, *PWT* posterior wall thickness, *LVDd* left ventricular end-diastolic diameter, *LVDs* left ventricular systolic diameter, *E* early diastolic filling velocity, *A* atrial filling velocity, *e′* mitral annulus velocity consisting of the early diastolic phase, *LVEDP* left ventricular end-diastolic pressure, *τ* isovolumetric relaxation time constant

## Discussion

The present study confirmed that left ventricular diastolic performance, LVEDP and *τ*, established by invasive techniques, correlate well with the E/e′lat, a non-invasive TDI parameter. However, the presence of RWMAlat on echocardiography weakened the correlation.

Assessing left ventricular diastolic performance is not only very important for the diagnosis of HFpEF, but also in that diastolic dysfunction contributes to heart failure with reduced systolic function. However, since “diastolic performance” is the product of a complex interaction of different factors in the diastolic phase, no unified parameter of diastolic performance can be established. Therefore, the non-invasive assessment of left ventricular diastolic performance is a far from easy task.

The European Society of Cardiology and the American College of Cardiology suggested a diagnostic strategy for HFpEF using E/e′ as the non-invasive diagnostic parameter [6, 22], because it closely correlates with the left ventricular filling pressure [17, 37, 38]. Passive filling of inflows into the left ventricle is determined by left ventricular stiffness and compliance. The earliest finding of diastolic dysfunction appears as a decrement of the ratio of early diastolic filling velocity (E) to atrial filling velocity (A), reflecting a relaxation delay of the left ventricular muscle. With further progression of diastolic dysfunction, increased LVEDP becomes evident. In clinical studies, it is clear that E/e′ correlates well with LVEDP [17, 19, 20, 39].

*τ*, which is the time constant of the descending limb of the left ventricular pressure curve, is a standard parameter of left ventricular relaxation; relaxation disorders result in the prolongation of *τ*. HFpEF patients with active relaxation disorders show prolonged *τ*, and *τ* also correlates well with E/e′ [20].

Parameters of left ventricular diastolic performance established invasively, such as LVEDP or *τ*, and non-invasively measured E/e′ are clearly assessed by different methodologies. Invasive parameters are obtained from the measurement of left ventricular pressure during isovolumetric relaxation or the rapid filling phase, whereas the E/e′ ratio is obtained by measuring the mitral annular velocity during the rapid filling phase. These differences influence the correlation between invasive parameters and the value of E/e′. It has been reported that various clinical features contribute to decreasing the strength of this correlation [19, 39]. Ommen et al. [17] pointed out that the correlation coefficient between E/e′ and LVEDP varies depending on the LVEF. Another report suggested the possibility that underlying cardiac conditions influence the correlation between E/e′ and LVEDP, recognized in differences between primary and secondary mitral regurgitation [24]. The correlation between E/e′ and *τ* might be less stable under certain clinical conditions, but this remains controversial [17, 20, 40–42].

RWMA is regarded as one of the most influential factors contributing to the magnitude of E/e′, because of the characteristics of the measurement methods. The present study showed that it is RWMAlat that worsens the correlation between invasive parameters and E/e′lat. We obtained values of E/e′ using a method whereby e′ was measured from the early relaxation velocity of lateral mitral annulus in an apical 4-chamber view. It has been reported that E/e′ measured at the septal mitral annulus correlates better with LVEDP or *τ* than when it is measured at the lateral mitral annulus [17]. On the other hand, one report suggested that E/e′lat correlates better with the LV filling pressure than when measured at the septal mitral annulus in cases with RWMA due to previous myocardial infarction [43]. These investigators recommended using E/e′lat in cases with RWMA. However, RWMAlat would be expected to have no small impact on the magnitude of E/e′lat, which we confirmed using the correlation with the invasive parameters LVEDP and *τ*. Our data suggest that the diagnostic value of E/e′lat for HFpEF is lower in patients with RWMAlat than those without. RWMA may, thus, decrease the accuracy of E/e′ for the diagnosis of HFpEF. We emphasize the necessity of using two E/e′ measurement methods (between the lateral and septal wall) depending on the clinical situation. At least in cases with RWMAlat, our data suggest that E/e′lat has low diagnostic value in the prediction of left ventricular diastolic performance. If the dissociation between lateral and septal value is shown, the value measured at the site without RWMA has higher credibility.

There are major limitations to this retrospective study. First, fluid-filled pigtail catheters were used in the pressure measurements, even though they are inferior in frequency response characteristics to a pressure-conductance catheter. Using the latter might result in better correlations of invasive parameters with E/e′. The time interval between echocardiographic assessment and invasive measurement is also a major concern. Moreover, the RWMAlat group tended to have lower systolic function, so it cannot be excluded that differences in baseline characteristics contribute to the weaker correlation in patients with RWMAlat.

Significant mitral regurgitation has a possibility to impact on the magnitude of E/e′. Though the present study included only one case (0.3 %) with severe mitral regurgitation, this impact on our outcome is thought to be small.

The left ventricular filling pressure or average left atrium pressure were not evaluated as invasive parameters of diastolic performance. These parameters might contribute to provide stronger evidence.

In conclusion, the E/e′lat ratio obtained by TDI is well correlated with parameters of left ventricular diastolic performance obtained by invasive techniques. However, E/e′lat might not reflect diastolic performance accurately in cases with RWMAlat. A careful assessment of E/e′lat is required in such patients for the prediction of diastolic performance.
